# Supplementary material for: Single-shot magnon interference in a magnon-superconducting-resonator hybrid circuit
Source: Nat Commun. 2025 Apr 17;16:3649. doi: 10.1038/s41467-025-58482-2 (PMC12006324; doi:10.1038/s41467-025-58482-2)
Supplement: Supplementary file 1 — Supplementary Information [file 41467_2025_58482_MOESM1_ESM.pdf]

# Supplementary Materials for

## Single-shot magnon interference in a magnon-superconducting-resonator hybrid circuit

Moojune Song, Tomas Polakovic, Jinho Lim, Thomas W. Cecil, John Pearson, Ralu Divan, Wai-Kwong Kwok, Ulrich Welp, Axel Hoffmann, Kab-Jin Kim, Valentine Novosad, and Yi Li

**Outline**

1. **Resonator mode for dispersive magnon-photon coupling**
2. **Rabi-like oscillation: frequency broadening from the pulse excitation**
3. **Theoretical derivation of magnon interference with two pulses**
4. **Two-pulse interference for $\boldsymbol{\delta = -g_{mm}}$**
5. **Frequency drift in the Fast Fourier Transform of magnon interference**
6. **Theoretical solution of magnon-photon-magnon hybrid dynamics and photon participation ratio**
7. **Frequency dependence of noise background**
8. **Arbitrary final magnon states**

## 1. Resonator mode for dispersive magnon-photon coupling.

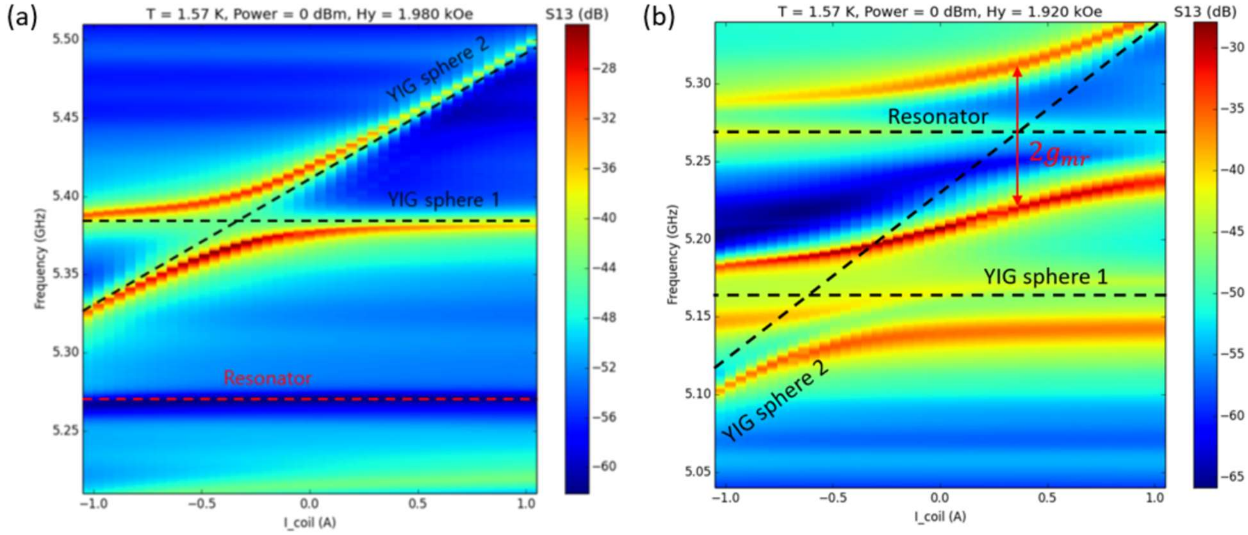


**Figure S1** VNA power transmission spectra measured at (a) $\boldsymbol{\mu_0 H_B = 0.198}$ T, and (b) $\boldsymbol{\mu_0 H_B = 0.192}$ T, showing the resonator mode at 5.27 GHz for dispersive magnon-photon coupling. (a) shows the location of the resonator when detuned from the magnon modes of the two YIG spheres, and (b) shows the magnon-photon coupling strength $\boldsymbol{g_{mr}}$.

Figure S1 shows the VNA power transmission spectra measured at (a) $\mu_0 H_B = 0.198$ T, and (b) $\mu_0 H_B = 0.192$ T. In (a), the resonator mode is located at 5.27 GHz, which mediates dispersive magnon-magnon coupling. We note that the resonator mode at 5.27 GHz is not the resonator mode which couples to the magnon modes the most strongly, or shows the best Q-factor. The resonator mode that couples the most strongly to the magnon modes is located at ~3.8 GHz, similar to our prior work [PhysRevLett.128.047701]. The reason that we did not choose the vicinity of 3.8 GHz is that, the background transmission is much higher at a lower frequency and the signal-to-background ratio is low. This will lead to large noise level for the time-domain pulse microwave experiments. Since the magnon-magnon interference is conducted in the dispersive magnon-photon coupling regime, the magnon relaxation process is not very sensitive to the damping of the resonator mode.

In (b), we repeat the measurement at a lower field where the magnon mode of one YIG sphere can be tuned by the local NbTi coil (diagonal dashed line) to cross the resonator mode at 5.27 GHz. From the magnon-photon anticrossing gap at $I_{\text{coil}} = +0.4$ A, we can extract the magnon-photon coupling strength as $g_{mr}/2\pi = 46$ MHz. This resonator mode provides the main contribution to the dispersive magnon-magnon coupling in the main text, because it is the closest to the two degenerate magnon modes at $\mu_0 H_B = 0.2$ T ($\omega_{m1}/2\pi = \omega_{m2}/2\pi = \omega_m/2\pi = 5.405$ GHz).

## 2. Rabi-like oscillation: frequency broadening from the pulse excitation.

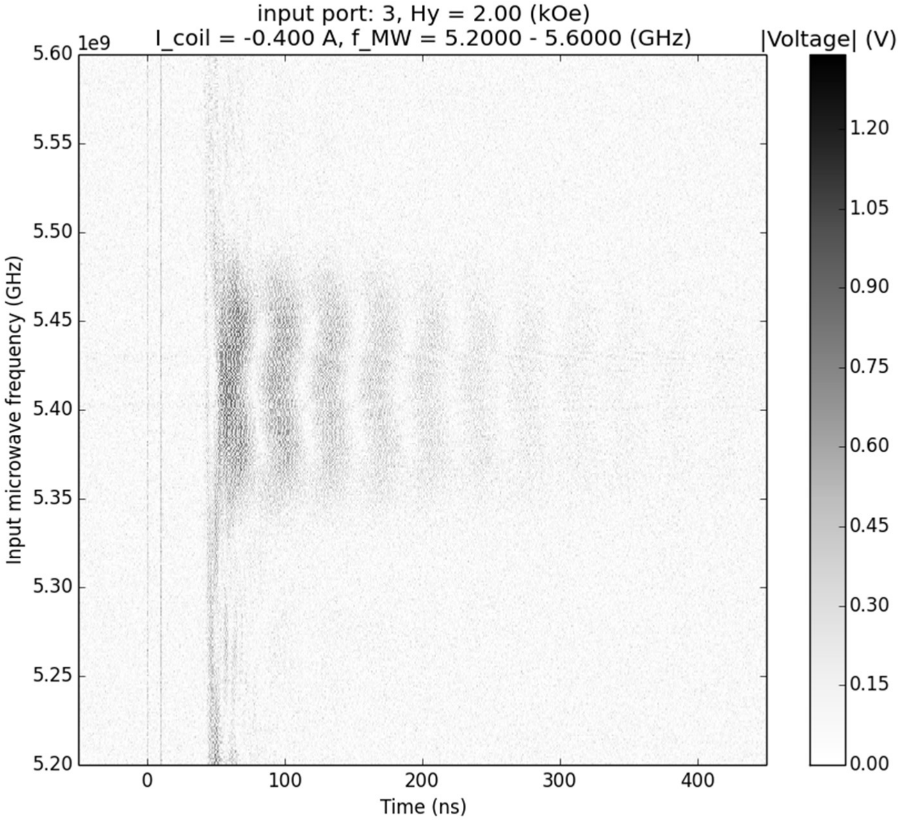


**Figure S2** Pulse excitation of Rabi-like oscillation between the two YIG spheres at different pulse frequencies, with $\mu_0 H_B = 0.2$ T and $I_{coil} = -0.4$ A where $\omega_{m1}/2\pi = \omega_{m2}/2\pi = 5.405$ GHz.

In Fig. 2(b) of the main text, we use a microwave pulse with a frequency of $\omega_m/2\pi = 5.405$ GHz to excite the Rabi-like oscillation between the two YIG spheres. Here $\omega_0$ is equal to the magnon frequency of the two individual YIG spheres ($\omega_m = \omega_{m1} = \omega_{m2}$). Fig. S2 shows the measured time traces for different pulse microwave frequencies. Rabi-like oscillation can be efficiently excited when the pulse frequency deviates from 5.405 GHz by about $\pm 0.05$ GHz. Note that the magnon mode linewidth is around 1 MHz and is much narrower than the frequency broadening from the pulse width, so the incident microwave pulse needs to be in the range of $5.405 \pm 0.001$ GHz in order to efficiently couple with the magnon mode. This excitation broadening is mainly due to the finite pulse width (10 ns), which leads to a frequency domain broadening of 1/10 ns ~0.1 GHz as confirmed by experiment. The period of the double-lobed pattern does not match with the eigenfrequencies of the two hybrid modes (5.390 GHz and 5.420 GHz) and is most likely due to the interaction between the YIG sphere and the broadened spectrum of the microwave pulse.

## 3. Theoretical derivation of magnon interference with two pulses

Here we derive the magnon interference with two consecutive microwave pulses in a hybrid magnon-magnon system. For the two magnon resonators $\overrightarrow{m_1}$ and $\overrightarrow{m_2}$, we take the assumption that they have the same eigenfrequency $\omega_0$ and are coupled with a strength of $g_{mm}$. The two hybrid eigenmodes $\overrightarrow{m_+}$ and $\overrightarrow{m_-}$ denote the in-phase (+) and out-of-phase (-) magnon resonance combinations and have eigenvalues of $\omega_m \pm g_{mm}$. The two eigenvectors can be expressed as:

$$\overrightarrow{m_+}(t) = \frac{1}{\sqrt{2}}(\overrightarrow{m_1} + \overrightarrow{m_2})e^{-i(\omega_m+g_{mm})t} \tag{S1}$$

$$\overrightarrow{m_-}(t) = \frac{1}{\sqrt{2}}(\overrightarrow{m_1} - \overrightarrow{m_2})e^{-i(\omega_m-g_{mm})t} \tag{S2}$$

At t = 0, a microwave pulse drives $\overrightarrow{m_1}$ from zero to an amplitude of "1", and the amplitude of $\overrightarrow{m_2}$ is still zero. The initial state can be decomposed to the superposition of $\overrightarrow{m_+}(t)$ and $\overrightarrow{m_-}(t)$:

$$\overrightarrow{m_{total}}(t=0) = \frac{1}{\sqrt{2}}[\overrightarrow{m_+}(t=0) + \overrightarrow{m_-}(t=0)] \tag{S3}$$

After a delay time $\Delta\tau$, the second pulse is applied to $\overrightarrow{m_1}$ again. However, note that the phase of the microwave pulse is locked to the synthesizer, there is also a phase delay between the second microwave pulse and the first microwave pulse as $\Delta\varphi = \omega\Delta\tau$ where $\omega$ is the microwave frequency. The time-dependent hybrid magnon state after the second pulse can be expressed as:

$$\overrightarrow{m_{total}}(t) = \frac{1}{\sqrt{2}}[\overrightarrow{m_+}(t) + \overrightarrow{m_-}(t)] + \frac{1}{\sqrt{2}}[\overrightarrow{m_+}(t-\Delta\tau) + \overrightarrow{m_-}(t-\Delta\tau)]e^{-i\omega\Delta\tau} \tag{S4}$$

In Eq. (S4), the first term comes from the first pulse at $t = 0$ and has the same form as in Eq. (S3). The second term comes from the second pulse at $t = \Delta\tau$, along with a phase lag $e^{-i\omega\Delta\tau}$ from the microwave. Eq. (S4) can be rewritten as:

$$\overrightarrow{m_{total}}(t) = \frac{1}{\sqrt{2}}\overrightarrow{m_+}(t)(1 + e^{-i(\delta-g_{mm})\Delta\tau}) + \frac{1}{\sqrt{2}}\overrightarrow{m_-}(t)(1 + e^{-i(\delta+g_{mm})\Delta\tau}) \tag{S5}$$

Here $\delta = \omega - \omega_m$ is the frequency detuning between the microwave and the magnon mode. The hybrid magnon state $(m_+, m_-)$ can be calculated from Eq. (S5):

$$m_+ = \left|1 + e^{-i(\delta-g_{mm})\Delta\tau}\right| = 2\left|\cos\frac{(\delta - g_{mm})\Delta\tau}{2}\right| \tag{S6}$$

$$m_- = \left|1 + e^{-i(\delta+g_{mm})\Delta\tau}\right| = 2\left|\cos\frac{(\delta + g_{mm})\Delta\tau}{2}\right| \tag{S7}$$

Eqs. (S6-7) recover Eq. (2) of the main text.

## 4. Two-pulse interference for $\delta = -g_{mm}$

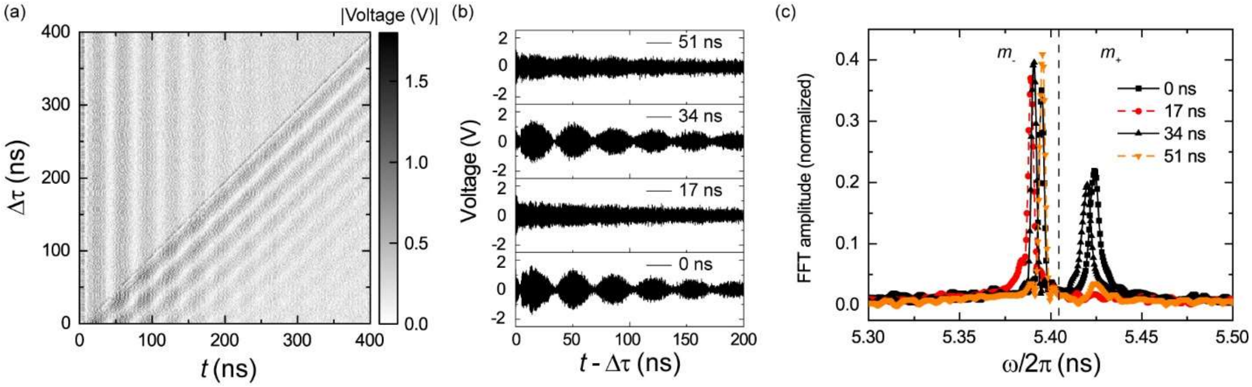


**Figure S3** (a) Time traces of the output signals for different $\Delta\tau$ with two-pulse excitations, measured at $\omega = \omega_0 - g_{mm}$. (b) Individual time traces of (a) at $\Delta\tau = 0$, 17, 34 and 51 ns, with the time axis shifted by $\Delta\tau$, starting right after the second pulse. (c) FFT spectra of the time traces from (b), showing alternating final states between (0,2) and (2,2).

In Figure S3, we show the two-pulse interference results for $\omega = \omega_m - g_{mm}$ or $\delta = -g_{mm}$, as a compliment to the Fig. 3 of the main text for $\omega = \omega_m$ and $\omega = \omega_m + g_{mm}$. The results show that the final magnon state $(m_+, m_-)$ oscillates between (0,2) and (2,2), which provide a new pattern of magnon state control.

## 5. Frequency drift in the Fast Fourier Transform (FFT) of magnon interference

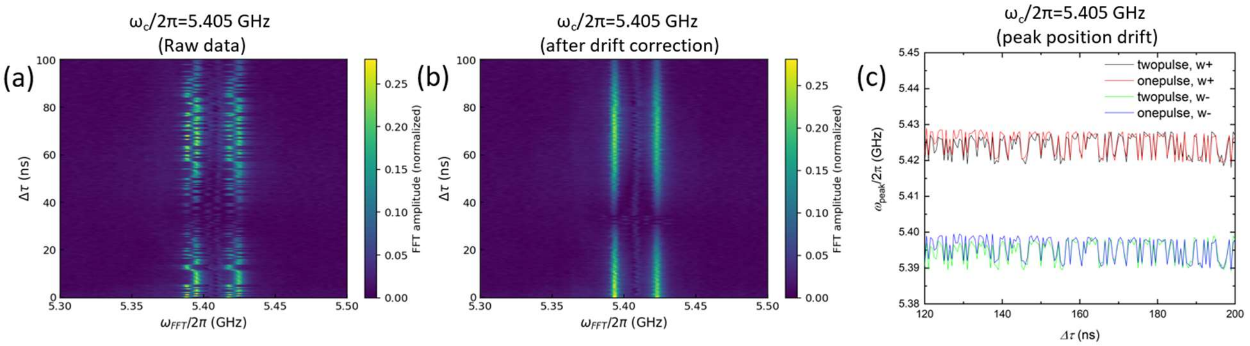


**Figure S4** (a) FFT color map of 2-pulse interference as a function of $\Delta\tau$ for $\omega_c/2\pi = 5.405$ GHz, showing random frequency drift by integers of 7.5 MHz. (b) FFT color map of (a) after frequency offset. (c) Evolution of FFT peak positions as a function of $\Delta\tau$, extracted from (a). The peak evolution of 1-pulse Rabi-like oscillation is also plotted, which overlap with the 2-pulse interference.

In Fig. 4(c) and (d) of the main text, the FFT color maps have undergone a frequency drift correction, which will be discussion in this section. In Figure S4, we show the FFT color map without frequency drift correction. The peak positions exhibit a random drift by a constant of 6 MHz (different Δτ means different measuring scans). For those drifted FFT spectra, both $\omega_+$ and $\omega_-$ peaks will drift together by the same amount, without changing their difference. Here we exclude the possibility of magnon frequency drift because the VNA measurements show stable magnon-magnon anticrossing along with narrow magnon linewidth of ~1 MHz. It is also unlikely to be from the pulse generation, because the pulse frequency broadening is ~ 100 MHz (see Section 2 in the Supplemental Materials) and is much larger than 6 MHz. The drift may come from an

artifact in real-time sampling. Since the oscilloscope does not have an infinite sampling rate (20 GSa/s), and the time traces has a finite length (0 – 400 ns), the FFT may lead to some irregular fluctuation.

In order to correct the frequency drift, we shift the entire FFT spectrum by -6 MHz when the $\omega_-$ mode frequency is more than 3 MHz away from 5.390 GHz. This yields Fig. S4(b), which is identical to Fig. 4(c) of the main text and shows no frequency drift for both $\omega_+$ and $\omega_-$ mode. Because the corrected FFT color map agrees with the theoretical prediction, supporting the coherent interaction from the hybrid $\omega_+$ and $\omega_-$ modes. We note that a weak peak is present in the middle of the $\omega_+$ and $\omega_-$ modes at 5.405 GHz, which may come from a finite cross talk between the two vertical antenna.

In Fig. S4(c), we plot the peak position of the $\omega_+$ and $\omega_-$ mode for $\omega_c/2\pi = 5.405$ GHz for both one-pulse (from 0 ns to Δτ) and two-pulse time traces (from Δτ to 400 ns). For Δτ in the range of 120 ns to 200 ns, they coincide with each other. This shows that the frequency drift is unrelated to two-pulse magnon interference and exists even with one-pulse excitation. For Δτ below 120 ns, since the length of the time traces are too short, the FFT spectra show an increasing error, but the drifting always happens at the same time for one-pulse and two-pulse time traces.

## 6. Theoretical solution of magnon-photon-magnon hybrid dynamics and photon participation ratio

In order to quantify the participation ratio of microwave photons during photon-mediated dispersive magnon-magnon coupling, we analytically solve the eigenfrequencies, eigenvectors, and their time evolutions from the equation of motion (assuming a sinusoidal time evolution $\sim e^{i\omega t}$):

$$\frac{d}{dt}\begin{pmatrix} a \\ m_1 \\ m_2 \end{pmatrix} = i\begin{pmatrix} \omega_r & g_{mr} & g_{mr} \\ g_{mr} & \omega_m & 0 \\ g_{mr} & 0 & \omega_m \end{pmatrix}\begin{pmatrix} a \\ m_1 \\ m_2 \end{pmatrix} \quad \text{(S8)}$$

where the magnon eigenfrequencies and magnon-resonator coupling strengths of the two YIG spheres are assumed to be identical ($\omega_m$ and $g_{mr}$) for simplicity. Here the vector $(a, m_1, m_2)$ gives the amplitudes of photon, magnon 1, and magnon 2 in the dynamics.

The eigenfrequencies can be solved from the following equation:

$$\begin{vmatrix} \omega_r - \omega & g_{mr} & g_{mr} \\ g_{mr} & \omega_m - \omega & 0 \\ g_{mr} & 0 & \omega_m - \omega \end{vmatrix} = 0 \quad \text{(S9)}$$

The 3 eigenfrequency solutions are:

$$\omega_0 = \omega_m \quad \text{(S10)}$$

and

$$\omega_\pm = \frac{\omega_r + \omega_m}{2} \pm \sqrt{\left(\frac{\omega_r - \omega_m}{2}\right)^2 + 2g_{mr}^2} \quad \text{(S11)}$$

Next, with the 3 eigenfrequencies, we can solve the eigenvectors from Eq. (S8), as:

$$\vec{v}_0 = \begin{pmatrix} 0 & 1 & -1 \end{pmatrix} \quad \text{(S12)}$$

and

$$\vec{v}_\pm = \begin{pmatrix} 1 & -\frac{g_{mr}}{\omega_m - \omega_\pm} & -\frac{g_{mr}}{\omega_m - \omega_\pm} \end{pmatrix} \quad \text{(S13)}$$

Next, we calculate the participation ratio of photon dynamics during dispersive magnon-magnon coupling. Assuming an initial state of (0, 1, 0) after the excitation of the first pulse which only excite the magnons in YIG sphere 1 ($m_1 = 1$, $a = m_2 = 0$), we can project the initial state to the three eigenvectors:

$$(0 \quad 1 \quad 0) = A\vec{v}_0 + B_+\vec{v}_+ + B_-\vec{v}_- \tag{S14}$$

The solution is:

$$A = \frac{1}{2} \tag{S15}$$

$$B_+ = -B_- = \frac{g_{mr}}{\omega_- - \omega_+} \tag{S16}$$

The final time evolution of the hybrid system with an initial state of (0, 1, 0) can be expressed as:

$$\vec{v}(t) = A\vec{v}_0 e^{i\omega_0 t} + B_+\vec{v}_+ e^{i\omega_+ t} + B_-\vec{v}_- e^{i\omega_- t} \tag{S17}$$

In particular, we show the time evolution of the microwave photon component in $\vec{v}(t) = (a(t), m_1(t), m_2(t))$, as:

$$\begin{aligned} a(t) &= \frac{g_{mr}}{\omega_- - \omega_+}\left(e^{i\omega_+ t} - e^{i\omega_- t}\right) \\ &= -\frac{i g_{mr} e^{i\frac{\omega_r+\omega_m}{2}t}}{\sqrt{\left(\frac{\omega_r - \omega_m}{2}\right)^2 + 2g_{mr}^2}} \sin(t \cdot \sqrt{\left(\frac{\omega_r - \omega_m}{2}\right)^2 + 2g_{mr}^2}) \end{aligned} \tag{S18}$$

The participation ratio of microwave photons is defined as the amplitude ratio between $a(t)$ and $m_1(t)$ or $m_2(t)$. Since the latter is one from the initial condition, the participation ratio is:

$$r = \frac{g_{mr}}{\sqrt{\left(\frac{\omega_r - \omega_m}{2}\right)^2 + 2g_{mr}^2}} \tag{S19}$$

One can see that $r$ is the ratio between $g_{mr}$ and $\sqrt{\left(\frac{\omega_r-\omega_m}{2}\right)^2 + 2g_{mr}^2}$. This describes how the microwave resonator mediates the dispersive magnon-magnon coupling, with a frequency which is detuned from the magnon frequency. Since $|\omega_c - \omega_m|$ is usually much larger than $g_{mr}$, the microwave resonator keeps a much lower photon occupation number compared with magnon occupation number, but pumps microwave photons with a much faster rate compared with the magnon-magnon transduction rate $g_{mr}$. If we take the values from the main text, $\frac{g_{mr}}{2\pi} = 46$ MHz, $\frac{\omega_r}{2\pi} = 5.27$ GHz, and $\frac{\omega_m}{2\pi} = 5.405$ GHz, we get $r = 0.25$. Below we plot in Figure S5 the time evolution of the amplitude, $a(t)$, $m_1(t)$ and $m_2(t)$, using the parameters above.

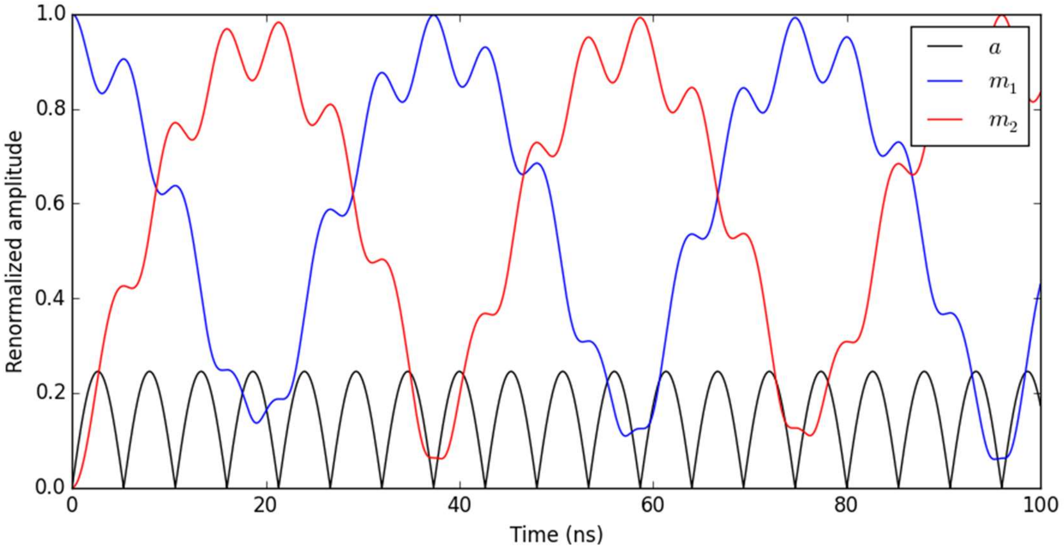

**Figure S5** Time evolution of $\boldsymbol{a(t)}$, $\boldsymbol{m_1(t)}$ and $\boldsymbol{m_2(t)}$, which are numerically calculated from Eq. (S17).

As shown in the plot, the amplitudes of $m_1$ and $m_2$ do not evolve as a perfect sinusoidal function, but with ripples which correspond to the energy exchange with the microwave photons in the superconducting resonator.

We discuss the impact of the finite photon participation ratio on the experiments demonstrated in the paper.

First, the impact on the Rabi-like oscillation (Fig. 2 of the main text) is very small, because the total power in the photon state is $r^2 = 6.25\%$, and the rest of the power $(1 - r^2 = 93.75\%)$, meaning that the energy is mostly relaxed in the form of magnons. In addition, the resonator mode at 5.27 GHz has a linewidth of 2 MHz. Compared with the magnon damping rate of 1 MHz in our experiment, this will increase the total energy relaxation rate by (2 MHz-1 MHz)* 6.25%=0.0625 MHz, which is negligible in our measurements.

Second, there could be an impact on the 2-pulse magnon interference, because upon the application of the second microwave pulse, part of the energy might be stored as the photon state and the phase of magnon state can be slightly disturbed. However, in the $\Delta\tau$ evolution, the impact on the magnon decoherence time $T_2$ should be limited because the energy storage in the photon state oscillates, instead of builds up, with $\Delta\tau$, and will only shift the Ramsey-like interference result by a small constant. The shift will mix with the noise background.

Last, for the multi-pulse magnon interference, the impact might become more complicated. This is because the excitation stored in the microwave photon state does not keep a fixed phase relationship with the magnon excitation and can add up as a function of pulse number. This may explain why the interference result deviates from the theoretical prediction as the pulse number increases.

## 7. Frequency dependence of noise background

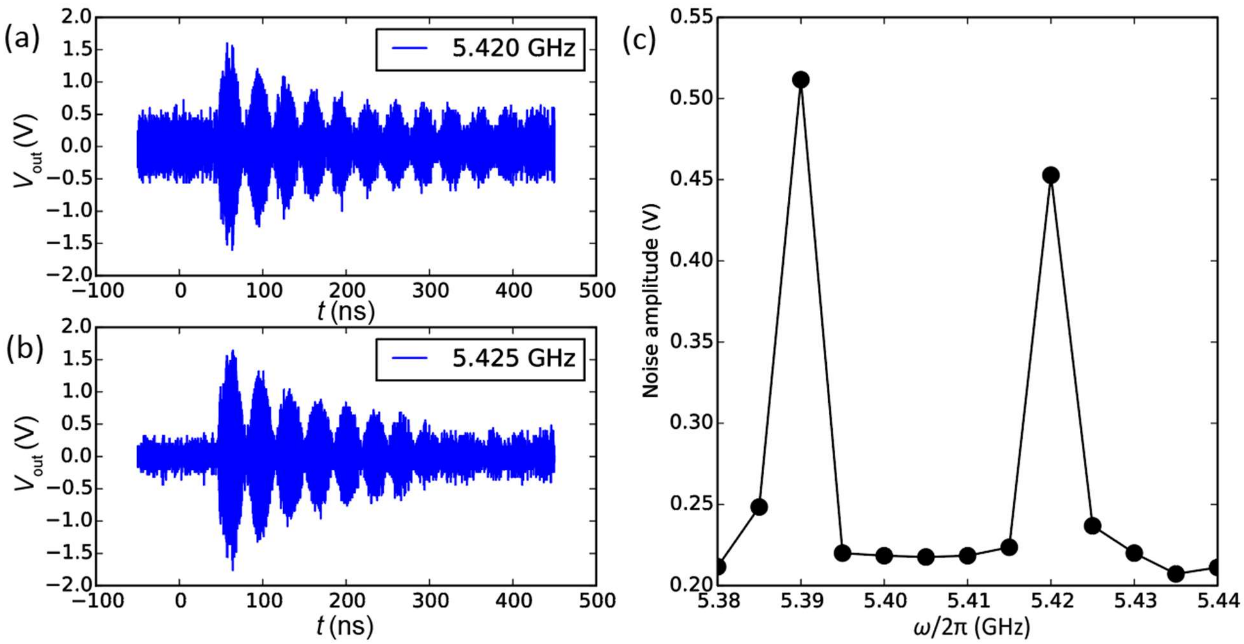


**Figure S6** (a-b) Rabi oscillation amplitudes and noise background with the pulse carrier frequency at (a) 5.420 GHz and (b) 5.425 GHz measured at $\boldsymbol{\mu_0 H_B = 0.2}$ T and $\boldsymbol{I_{coil} = -0.4}$ A, showing a high noise background at 5.420 GHz. (c) Extracted noise amplitude by averaging 10 local maxima between -50 and 0 ns, showing two peaks at 5.390 and 5.420 GHz.

During the time-domain pulse magnon measurements, we notice that the noise background has a frequency dependence. For example, as shown in Fig. S6 (a) and (b), the Rabi-like oscillation shows similar amplitude and exponential decay time at 5.420 GHz and 5.425 GHz, but their noise amplitudes (from -50 ns to +40 ns) are quite different. In Fig. S6(c) we show the extracted noise amplitude as a function of pulse carrier frequency. Two peaks are clearly shown at 5.390 GHz and 5.420 GHz. They coincide with the eigenfrequencies of the two hybrid magnonic mode ($\omega_0 \pm g_{mm}$). We think it is likely due to some feedback interaction between the hybrid resonator circuit and the amplifier, because the amplifier can amplify the high-Q-factor resonance signal much more efficiently. It is also possible that the noise maximum is simply the characteristics of the amplifier. In either case, this is an artifact of the microwave circuit. In the main text, this artifact is responsible for the slower decay of one-pulse Rabi-like oscillation in Fig. 3(d) compared with Fig. 3(b), as well as the offset between the green and black traces in Fig. 4(i).

## 8. Arbitrary final magnon states

Here we calculate the required $\Delta\tau$ and $\delta$ for an arbitrary hybrid magnon state ($m_+$, $m_-$)=( $a$, $b$) where $0 \leq m_+, m_- \leq 2$. From Eqs. (S10) and (S11), we have:

$$a = 2\left|\cos\frac{(\delta - g_{mm})\Delta\tau}{2}\right| \quad \text{(S11)}$$

$$b = 2\left|\cos\frac{(\delta + g_{mm})\Delta\tau}{2}\right|$$

The solutions of Eq. (S11) for $\Delta\tau$ and $\delta$ are:

$$\Delta\tau = \frac{\cos^{-1}\frac{b}{2} - \cos^{-1}\frac{a}{2}}{g_{mm}}$$

$$\delta = g_{mm}\frac{\cos^{-1}\frac{b}{2} + \cos^{-1}\frac{a}{2}}{\cos^{-1}\frac{b}{2} - \cos^{-1}\frac{a}{2}} \tag{S12}$$

From Eq. (S11), $a/2$ and $b/2$ can only take values between 0 and 1, so $\cos^{-1}(a/2)$ and $\cos^{-1}(b/2)$ can take either the positive value between 0 and $\pi/2$, or the negative value between $-\pi/2$ and 0. For the convenience in Eq. (S12), we can take a positive $\cos^{-1}(b/2)$ and a negative $\cos^{-1}(a/2)$, so that $\Delta\tau$ will take the value between 0 and $\pi/g_{mm}$, and $\delta$ will take the value between 0 and $g_{mm}$. In another word, from Eq. (S12), any arbitrary hybrid magnon state ($m_+$, $m_-$)=( $a$, $b$) can be obtained for $0 \leq \Delta\tau \leq \pi/g_{mm}$ and $0 \leq \delta \leq g_{mm}$.
